# Migrasome, a migration-dependent organelle

**DOI:** 10.3389/fcell.2024.1417242

**Published:** 2024-06-06

**Authors:** Fuyong Zhang, Wendong Liu, Yunpeng Mao, Yuhao Yang, Chenxi Ling, Ya Liu, Feng Yao, Yunfang Zhen, Xiaodong Wang, Mincheng Zou

**Affiliations:** Department of Orthopaedics, Children’s Hospital of Soochow University, Suzhou, China

**Keywords:** migrasome, cell migration, physiological and pathological processes, intercellular communication, disease occurrence and development

## Abstract

Migrasomes are organelles produced by migrating cells that form on retraction fibers and are released during cell migration. Migrasomes are involved in physiological and pathological processes such as intercellular communication, cell homeostasis maintenance, signal transduction, disease occurrence and development, and cancer metastasis. In addition, methods and techniques for studying migrasomes are constantly evolving. Here, we review the discovery, formation process, regulation, and known functions of migrasomes, summarize the commonly used specific markers of migrasomes, and the methods for observing migrasomes. Meanwhile, this review also discusses the potential applications of migrasomes in physiological processes, disease diagnosis, treatment, and prognosis, and looks forward to their wider application in biomedicine. In addition, the study of migrasomes will also reveal a new perspective on the mechanism of intercellular communication and promote the further development of life science.

## 1 Introduction

Extracellular vesicles (EVs) are structures that are released by all cells into their cellular environment. They are encased in lipid bilayers and contain components in the cells that release them. As important communication tools between cells, extracellular vesicles participate in various physiological and pathophysiological processes. Migrasomes, which are formed during cell migration, have recently attracted much attention as a potential tool for cell communication. Migrasomes are organelles generated by migrating cells and vesicles formed on the retraction fibers (RF) of migrating cells (Ma et al., 2015). Migrasomes are organelles rather than extracellular vesicles (Ma et al., 2015). Although detached migrasomes are extracellular vesicles, the production of extracellular vesicles (the detached migrasome) is just one of many functions of the migrasomes that they can perform as part of the cell before shedding from the cell. In this article, we review the discovery process of migrasomes and summarize their formation process, what is known about their functions in intercellular communication and embryonic development, their role in physiological and pathological processes, and the methods for observing and studying migrasome movement. Possible future research areas and clinical applications of migrasomes are also proposed.

## 2 Discovery of migrasomes

Cell migration refers to the movement of cells after receiving migration signals or feeling the gradient of certain substances. Cell migration is a fundamental function of normal cells, a physiological process of proper body growth and development, and a universal mode of movement for live cells. Cell migration plays a role in embryonic development, angiogenesis, wound healing, immunological and inflammatory responses, atherosclerosis, cancer metastasis, and other processes (Kobayashi et al., 2003; Sahai, 2005; Coles et al., 2007; Friedl and Gilmour, 2009; Shaw and Martin, 2009; Weijer, 2009).

In 2015, Yu et al. accidently discovered that during the migration process of cells under the microscope, rapidly moving cells were lit up by fluorescent proteins and dragged behind them by a large number of long filamentous lines, called “retraction fibers” (RFs). The migrating cells will pull out many RFs behind them, and some small vesicles about 0.5μm–3 μm in diameter will be produced at the tips and intersections of these RFs. Because the formation of these vesicles is dependent on cell migration, they are named “migrasomes” (Ma et al., 2015). These vesicles also contain vesicles with a diameter of 50–100 nm (the number of vesicles ranges from 10 to 300), which were originally called pomegranate-like structures (PLS) because they resembled pomegranate (Yu and Yu, 2022). In the process of cell migration, the cell will continue to transport some intracellular substances to the migrasomes through the channel of the RFs, and then the cell will migrate away, the RFs will break, and the migrasomes will be released. Cellular contents such as vesicles and cytosol can be released from the cell by migrasomes and subsequently taken up by the extracellular space or surrounding cells.

The formation of migrasomes has a fixed pattern: an initial rapid growth, followed by a relatively stable period. Finally, the RFs are broken, the migrasomes separate, and the migrasomes and their contents, including cytosolic components and small vesicles, are released into the matrix, which can also be taken up and utilized by other cells. This migration-dependent release mechanism is termed “Migracytosis” (Ma et al., 2015).

The first systematic observation of migrasomes revealed that they form and carry intracellular substances during cell migration, and observed the whole process of migration-dependent release mechanism called “Migracytosis”. It lays a foundation for further study on the formation, release and function of migrasomes.

## 3 Molecular mechanisms of the bioformation process of migrasomes

**FIGURE 1:**
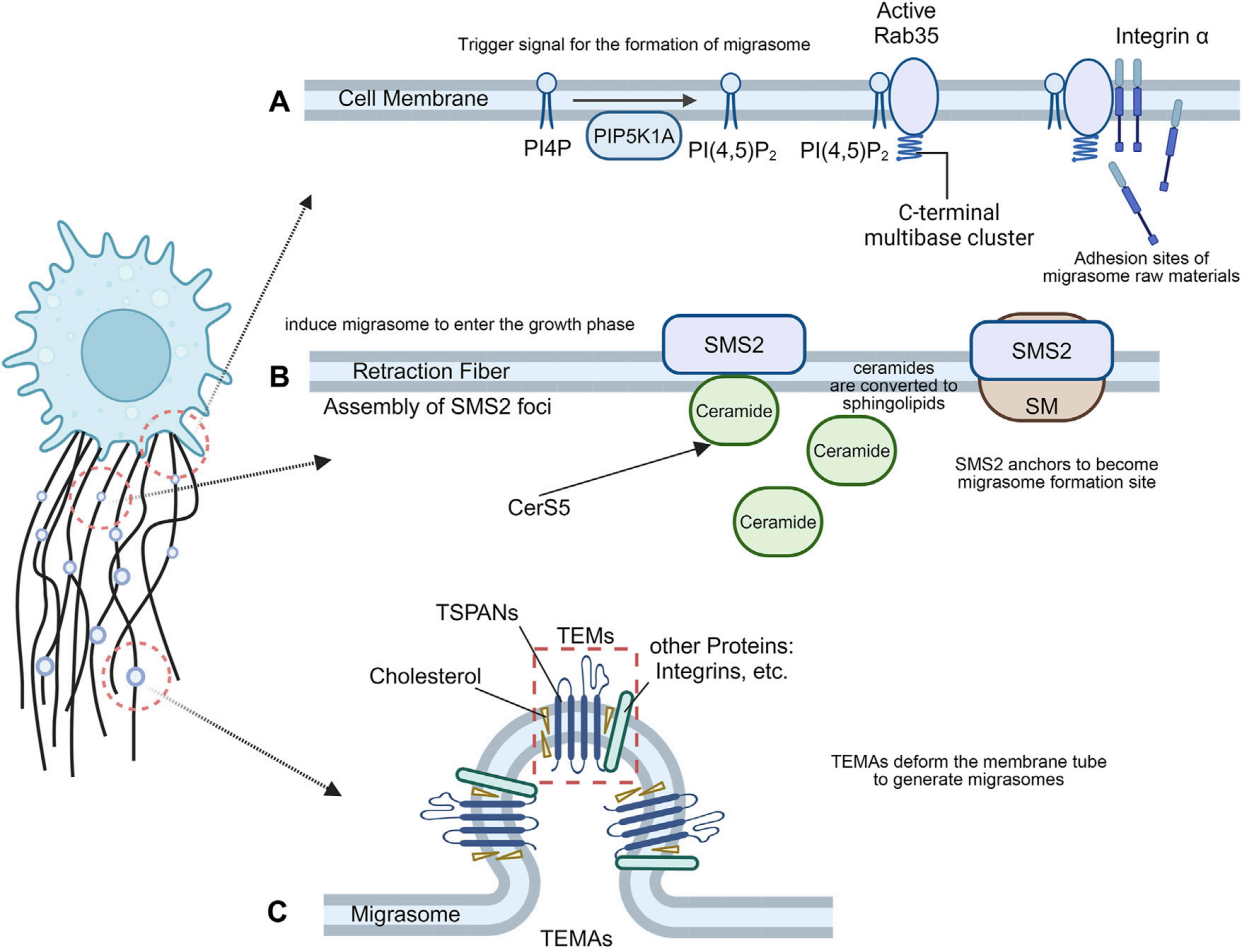
The formation of migrasomes. **(A)** Migrasome nucleation process. The recruitment of PIP5K1A and *de novo* synthesis of PI(4,5)P2 at the migrasome formation site may be the trigger signal for migrasome formation, and the interaction between active Rab35 and integrins creates the necessary adhesion sites for migrasome formation. **(B)** Migrasome maturation process. Cells migrate away, and the anchored SMS2 spot remains on the retraction filament as the migrasome formation site. At the SMS2 spot site, ceramide is converted into sphingolipin, which induces migrasome growth. **(C)** Migrasome expansion process. TSPAN4 can self-assemble or integrate with other transmembrane proteins and cytoplasmic proteins to form TEMs, which then aggregate into TEMAs to form a migrasome structure.

As mentioned above, the macroscopic formation of migrasome was observed under the microscope. Migrasomes are mainly derived from the ends and intersections of RFs in motile cells. Their production is closely related to cell motility and undergoes three key steps: nucleation, maturation, and expansion (Zhai et al., 2024) (Figure 1). In this section, we will further explore the specific mechanism of migrasome formation from the molecular level.

Migrasome formation is an active biogenesis process that is tightly regulated by signaling pathways, rather than a membrane shedding process where membrane debris is passively lost from the trailing edge of migrating cells (Ding et al., 2023). Phosphoinositol kinases have long been proposed to trigger migrasome formation (Zhen and Stenmark, 2023). PIP5K1A is recruited to the migrasome formation site to convert PI4P to PI(4,5)P2 and accumulate. Rab35, an evolutionarily conserved Rab GTPase located at endosomes and plasma membranes, has attracted much attention for its role in cytokinesis and cell migration (Klinkert and Echard, 2016). During cell division, PI(4,5)P2 hydrolysis is important for normal cytokinesis shedding as well as local remodeling of the F-actin cytoskeleton in intercellular bridges, which may be mediated by Rab35 (Dambournet et al., 2011). During the formation of migrasomes, PI(4,5)P2 recruits Rab35 to the migrasome formation site by interacting with the C-terminal multibase cluster of Rab35. Active Rab35 promotes migrasome formation by recruiting and concentrating integrin α5 at the migrasome formation site (Figure 1A). Taken together, the recruitment of PIP5K1A and *de novo* synthesis of PI(4,5)P2 at the migrasome formation site may be trigger signals for migrasome formation. Interactions between active Rab35 and integrins at the migrasome formation site prepare adhesion sites for molecules required for tetrasamectin dependent amplification for migrasome formation (Ding et al., 2023).

The plasma membrane contains a large proportion of sphingomyelin (SM) (Huitema et al., 2004). Sphingomyelin synthase (SMS) is the only enzyme in mammals that produces SM by transferring phosphocholine from phosphatidylcholine to ceramide (Taniguchi and Okazaki, 2021). Sphingolipid synthase 2 (SMS2) is primarily localized at the plasma membrane (Yeang et al., 2008), and primarily regulates the activity of plasma membrane SM (Huitema et al., 2004), directly or indirectly altering the biophysical properties of the membrane (Milhas et al., 2010). SM forms SM-rich microdomains on the cell membrane, which profoundly affect cell signaling, such as cell migration (Taniguchi and Okazaki, 2014). SMS2 is an essential protein for migrasome biogenesis, and the formation of sphingolipid catalyzed by SMS2 is enriched in migrasomes. Liang et al. revealed the important role of ceramide and sphingomyelin in the formation of migrasomes and showed that SMS2 forms a basement membrane surface junctional structure that predetermines where migrasomes grow (Liang et al., 2023). Cells migrate away, and the anchored SMS2 spot remains on the RFs and becomes the migrasome formation site. At the SMS2 spot position, ceramide is converted into sphingolipid, which induces the migrasome to enter the growth phase (Figure 1B). Knockdown of SMS2, Sgms2 genes, or treatment of cells with SMS2 inhibitors significantly reduced the number of migrasomes. The formation of migrasomes is initiated by the assembly of SMS2 foci at the leading edge of migrating cells (Liang et al., 2023).

TSPANs are a family of four transmembrane proteins that are present in every cell type (Rubinstein, 2011). Overexpression of TSPAN1, 2, 3, 4, 5, 6, 7, 9, 13, 18, 25, 26, 27 and 28 enhance migrasome formation, and among them TSPAN1, 2, 4, 6, 7, 9, 18, 27 and 28 have a strong effect (Huang et al., 2019). Each TSPANs has specific partners, including various integral proteins (Charrin et al., 2009). Tetraspanin 4 (TSPAN4) has previously been shown to be abundant in migrasome membranes (Ma et al., 2015), and required for migrasome formation (Huang et al., 2019). As one of the most potent tetraspanins to induce migrasomes, Tetraspanin4 (TSPAN4) and cholesterol play a key role in the formation of migrasomes. Yu et al. established three normal rat kidney (NRK) epithelial cell lines stably expressing different levels of TSPAN4 and green fluorescent protein (GFP) and found that TSPAN4 is one of the most effective tetraspanin proteins to induce migrasomes (Ma et al., 2015). TSPAN4 and cholesterol are required for migrasomes formation *in vivo* (Huang et al., 2019). Furthermore, an *in vitro* system mimicking the process of migrasome formation was able to demonstrate that TSPAN4 and cholesterol are sufficient to form migrasome-like structures (Huang et al., 2019). TSPANs can form TEMs by interacting and regulating related molecules at the membrane plane (Charrin et al., 2009). Like other members of the TSPAN family, TSPAN4 can self-assemble or integrate with other transmembrane proteins and cytoplasmic proteins to form tetraspanin-enriched microdomains (TEMs) (Huang et al., 2019). During migrasome generation, TEMs assembled by TSPAN4 can aggregate to form micron-level macrodomains (TEMAs), which eventually deform the membrane tube to generate migrasomes. The latter expanded into a large vesicular migrasome shape (Huang et al., 2019). Cell migration induces a high local enrichment of Tspan4 protein and cholesterol on RFs, thereby increasing the flexural stiffness of the membrane in the enriched region, resulting in the formation of a migrasome structure (Dharan et al., 2023) (Figure 1C). The primary role of Tspan4 is to stabilize the migrasome structure, while migrasome nucleation and the initial growth phase can be driven by membrane mechanical stress (Dharan et al., 2023). Knockdown of TSPAN4 reduced the formation of migrasomes (Lee et al., 2024).

In summary, three key steps are involved in the process of migrasome formation, in which molecules such as Rab35, SMS2 and TSPAN4 and their interactions with other molecules play important roles. An in-depth understanding of these molecular mechanisms will help to explore the functions and regulatory mechanisms of migrasomes.

## 4 Cytoskeletal compositions of migrasomes

It is well known that the cytoskeleton is a network of interconnected microtubules, microfilaments, and intermediate fibers. Using inhibitors of cytoskeletal components, researchers found the compositions of the migrasome includes F-actin, α-tubulin and vimentin intermediate filaments, which is a solid step to explore the cytoskeletal compositions of migrasomes.

3D rendering of the migrasomes revealed that they contained F-actin and α-tubulin, and analysis revealed that actin was present in the RFs and their associated migrasomes, whereas α-tubulin was restricted to the migrasomes (Deniz et al., 2023).

Aggregation of active Rab35 at the formation adhesion site has been observed during the nucleation step of migrasome formation. Rab35 is thought to act as a key regulator to regulate F-actin at the plasma membrane, thereby achieving its various cellular functions (Klinkert and Echard, 2016). The formation of migrasomes also depends on the polymerization of actin. When preventing the formation of branched actin networks (application of arp2/3 complex inhibitor), the formation of migrasomes was greatly reduced (Ma et al., 2015). The polymerization of actin could either inhibit the formation of migrasomes by inhibiting cell migration or, more likely, directly affect the formation of migrasomes structures.

Recent studies have shown that vimentin intermediate filaments interact and regulate cytoskeletal dynamics that drive cell motility (Battaglia et al., 2018). Vimentin controls actin stress fibers through RhoA and promotes cell migration (Jiu et al., 2017). Loss of vimentin results in defects in both persistence and speed of cell migration, producing fewer migrasomes than wild-type cells (Fan et al., 2022).

## 5 Cell migration affects migrasome formation

It is worth noting that the formation of migrasomes depends on cell migration. It has been established that the generation of PLS is premised on cell migration (Ma et al., 2015; Lu et al., 2020). In NRK cells stably expressing TSPAN4-GFP and in zebrafish embryonic cells, it was found that the number of migrasomes increased with the use of agonists that enhance cell migration and decreased with the use of inhibitors that suppress cell migration (Lu et al., 2020). These studies confirmed that the generation of migrasomes is dependent on cell migration. Treatment of cells with blebbistatin, a myosin II inhibitor that interferes with cell migration, inhibits migrasome formation (Wu et al., 2017).

The pattern of cell migration can also indirectly affect the formation of migrasomes. During non-persistent migration, cells formed fewer migrasomes due to the narrower tail of cells during turning, producing fewer RFs; In addition to motility persistence, cell migration speed limits migrasome formation by controlling the length of RFs.

The basic mechanism of intrinsic regulation of cell-directed migration is related to the Rho family of small GTPases and the integrin family of ECM receptors (Raftopoulou and Hall, 2004; Caswell and Norman, 2006).

As an important adhesion molecule in cell migration, Integrin mediates the mutual recognition and adhesion between cells and cells as well as between cells and extracellular matrix, and has the role of connecting the external action of cells with the internal structure of cells (Hynes, 2002). The correct pairing of integrins with extracellular matrix components is also a key factor in the formation of migrasomes. In contrast to the relatively low expression on RFs, ITGB1 (integrin β1) and ITGA5 (integrin α5) in the integrin family are relatively highly enriched in migrasomes and have been suggested as possible specific markers for migrasomes detection (Wu et al., 2017). By using siRNA to eliminate the levels of ITGA5 and TSPAN4, fewer RFs and migrasomes were observed in U87MG cells than in control cells (Lee et al., 2024).

In addition, high-throughput screening has identified Rho-associated kinase 1 (ROCK1) as a regulator of migrasome formation (Lu et al., 2020). By screening, the ROCK1 inhibitor SAR407899 was identified, which interferes with migrasome biogenesis but does not significantly reduce RFs formation, and is not cytotoxic or causes impaired cell proliferation (Lu et al., 2020). ROCK1 regulates cell adhesion to fibronectin (Bhadriraju et al., 2007), an important factor in regulating migrasome formation.

## 6 Methods and techniques for studying migrasomes

A common protocol for the purification and observation of migrasomes has been developed (Jiang D. et al., 2023): a) Detection and observation of migrasomes by fluorescence microscopy imaging; b) Purification of migrasomes from cultured cell lines and embryos by density gradient centrifugation; c) Characterization of migrasomes by electron microscopy imaging and biochemical analysis.

### 6.1 Microscopy and imaging methods used to visualize migrasome dynamics

Studying migrasomes relies heavily on imaging. Migrasomes were first observed by transmission electron microscopy (TEM) and scanning electron microscopy (Migrasomes, 2021). In 2015, Yu et al. used TEM to observe membrane-bound vesicle structures in the extracellular space surrounding NRK cells, with RFs attached at their base and finally identified these pomegranate-like structures as migrasomes (Ma et al., 2015). This indicates that observation under TEM remains the most reliable and definitive method for the identification of migrasomes.

Long-term high-speed high-resolution 3D imaging of deep tissues remains an unresolved systemic challenge. Serial 3D imaging by two-photon microscopy (TPM) can cause severe damage to *Drosophila* embryos. Using the same laser power, 2pSAM can continuously record the entire process for more than 17 h at high 3D imaging speeds and with higher hatching rates (Zhao et al., 2023), providing excellent opportunities to better understand how living systems organize and respond to different stimuli over longer time windows. However, for dense samples, the net imaging rate slows down (Zhao et al., 2023).

Small size (0.5–3 μm) and high 3D mobility make it difficult to visualize migrasome dynamics *in vivo*. Compared with TPM, digital adaptive optics scanning light-field mutual iterative tomography (DAOSLIMIT) exhibits better spatiotemporal resolution and lower phototoxicity. There have been reports that capture the entire process of migrasome biogenesis in mice (Wu et al., 2021). DAOSLIMIT allows *in vivo* observation of 3D subcellular dynamics on a millisecond scale for up to an hour, which is of great value for precise and complete observation of the activity of the studied migrating cells and the formation of migrasomes.

### 6.2 Markers of the migrasome model

In terms of markers for migrasome visualization, the transmembrane protein TSPAN4 is not unique to migrasomes, it is also localized to the plasma membrane and RFs (Ma et al., 2015), and therefore cannot be used to identify migrasomes. However, it has been identified as an imaging marker because it is highly enriched on migrasomes.

The pairing of integrins with their specific ECM chaperones is a determinant of migrasome formation, which may become an important principle in determining when and where migrasome is generated *in vivo* (Wu et al., 2017). Although TSPAN4 and integrins are highly enriched on migrasomes, especially integrin-GFP was rarely localized in RFs, while TSPAN4 protein was also detected in large quantities in RFs (Wu et al., 2017). Excellent integrin antibodies are commercially available, so they may become an important tool for the study of migrasomes *in vivo*.

Protein composition is only 27% identical between migrasomes and exosomes (Zhao et al., 2019). Four specific proteins present in the migrasomes were identified: NDST1 (bifunctional heparan sulfate N-deacetylase/N-sulfotransferase 1), PIGK (phosphatidylinositol glycan anchor biosynthesis, class K), CPQ (carboxypeptidase Q) and EOGT (EGF domain-specific O-linked N-acetylglucosamine transferase). They are enriched in migrasomes but are absent or barely detectable in exosomes and can be used as markers for the biochemical detection of migrasomes (Zhao et al., 2019).

## 7 Function and biological significance of migrasomes

**FIGURE 2:**
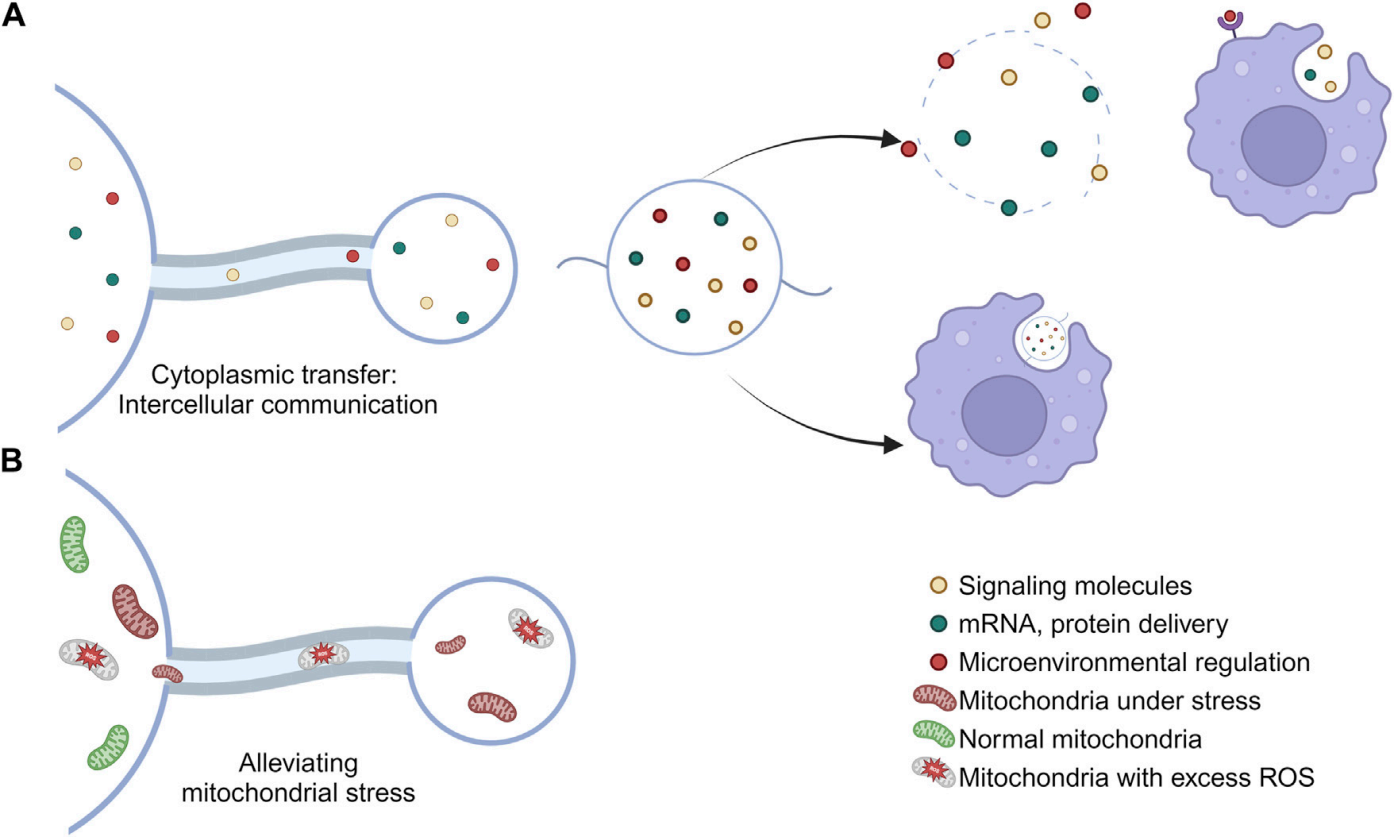
Physiological functions of migrasomes. **(A)** The migrasomes may contain mRNAs, proteins, cytokines, and other substances found in the cytoplasm of the cell body. When a cell migrates away, the migrasome will remain in place until it breaks down or is engulfed by other cells, thus carrying out the intercellular material transfer. **(B)** Migrasomes are involved in the maintenance of cell homeostasis and the removal of stress mitochondria.

In addition to participating in cell migration, migrasomes also have many important physiological functions (Figure 2). According to the relevant studies published so far, the physiological functions of migrasomes are mainly manifested as follows.

### 7.1 Maintenance of cell homeostasis

Migrating cells consume more energy than quiescent cells, so they may have higher respiration rates, ROS production, and mitochondrial stress load (Poole and Macleod, 2021). Therefore, migrating cells necessarily require additional mechanisms to mitigate this higher mitochondrial stress burden. Mitochondrial shedding contributes to mitochondrial quality control (Melentijevic et al., 2017). Migrasomes can regulate the quality of mitochondria, thereby maintaining intracellular mitochondrial homeostasis by clearing out damaged mitochondria (Jiao et al., 2021) and avoiding the adverse effects caused by the accumulation of damaged mitochondria, such as cytochrome C release from mitochondria and subsequent caspase activation (Jiang and Wang, 2004; Bock and Tait, 2020). This process is known as mitocytosis. Current studies have shown that there are two main ways to solve mitochondrial stress *in vivo*, called mitocytosis and mitophagy. mitocytosis is induced by mild mitochondrial stress. Notably, 10 μM CCCP (the dose used to induce mitophagy) induced only a small amount of mitocytosis. This may be explained by the fact that cells barely migrated in the presence of 10 μM CCCP (Jiao et al., 2021). Mitocytosis and mitophagy may serve as a two-gear system to maintain mitochondrial quality in migrating cells, with mitocytosis responsible for handling mild mitochondrial damage, which may occur frequently under physiological conditions, while mitophagy handles severe mitochondrial damage associated with pathological conditions (Jiao et al., 2021).

### 7.2 Migrasome-derived nanoparticles: intercellular communication

As a single-layer, releasable vesicular structure, researchers have proposed that migrasomes function as signaling organelles, providing specific biochemical information to neighboring cells (Jiang et al., 2019).

The active transfer of cytoplasm from the main body of the cell to the migrasomes was observed by GFP tracing (Ma et al., 2015), and therefore the migrasomes may contain mRNAs, proteins, cytokines, and other substances that are possessed in the cytoplasm of the cell body (Zhu et al., 2021). When a cell migrates away, the migrasome remains in place until it breaks down or is engulfed by other cells (Ma et al., 2015), thereby carrying out intercellular material transfer (Zhu et al., 2021).

In addition, small vesicles containing different numbers termed migrasome-derived nanoparticles (MDNPs), were observed in the migrasomes. Such nanoparticles are produced by the migrasome by self-rupture and release of internal vesicles by a process similar to cytoplasmic membrane budding (Ma et al., 2023). MDNP has a membrane structure with typical circular morphology and has characteristic markers of migrasomes. Interestingly, MDNP is also loaded with a large number of microRNAs that are distinct from those in migrasomes, which predicts additional functions for these released small vesicles. MDNP is present or released as a content of the migrasome. Some scholars have suggested that migrasomes are capable of carrying and releasing vesicular structures, even exosomes (Ma et al., 2015). Further studies are needed on the properties, structure, and composition of MDNP.

### 7.3 Signal integration between cells

The presence of chemokines, cytokines, growth factors, and other signaling molecules in migrasomes suggests that they have complex and special functions. Recent studies have shown that migrasomes play an important role in signal transduction in embryonic development, immune response, and cancer metastasis.

**FIGURE 3:**
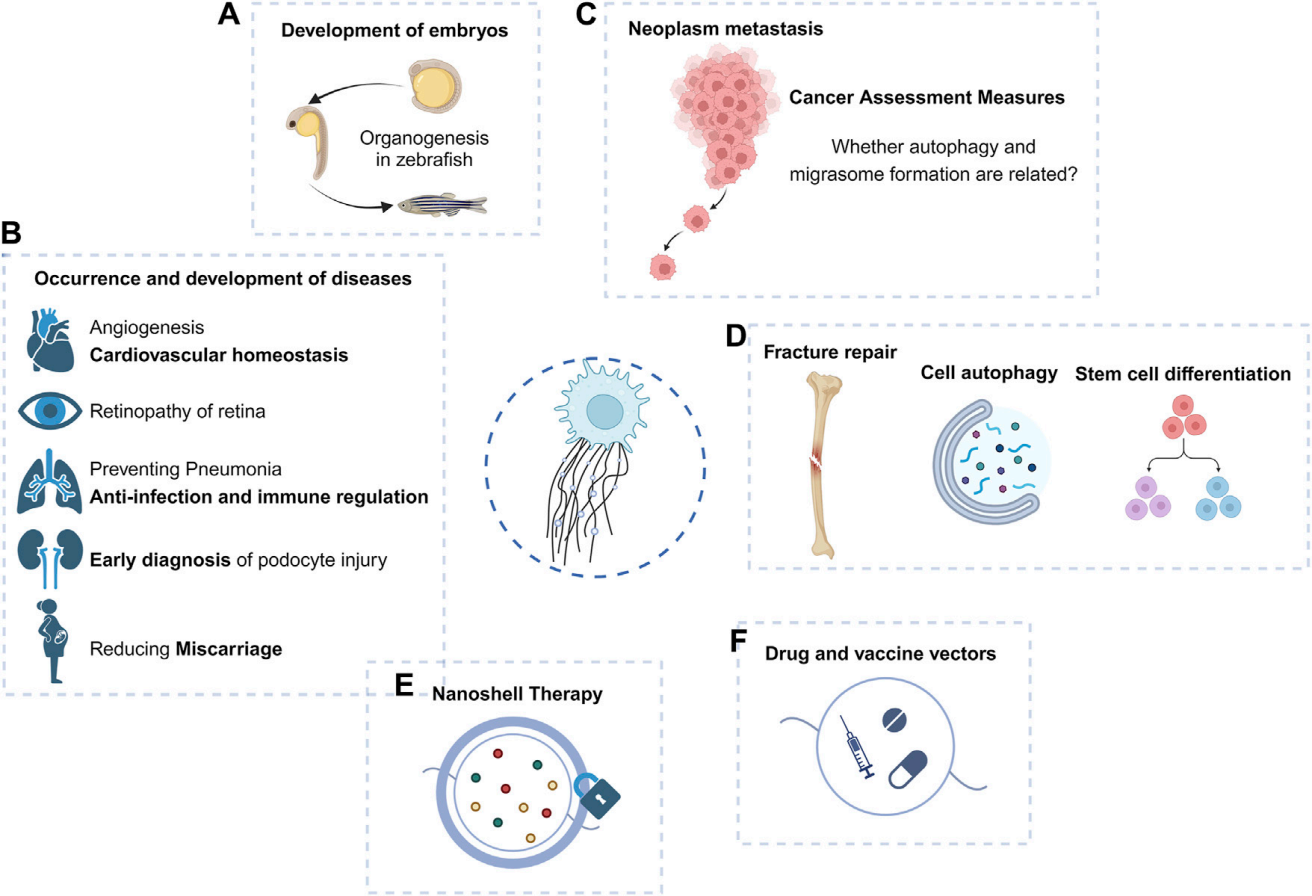
Macro function and application prospect of migrasomes. **(A–D)** Migrasomes have shown important roles in embryonic development, tumor migration, and disease occurrence and development. **(E,F)** Because of its role in intercellular communication, the prospect of migrasomes as drug carriers or therapeutic targets should not be underestimated.

Studies on the role of migrasomes in zebrafish embryonic development have found that migrasomes serve as membrane-coated carriers of signal molecules, which determine the spatial and temporal distribution of signal molecules, and thus play a new mechanism in regulating organ development (Figure 3A). Studies have found that when the production of migrasomes is blocked, zebrafish will show abnormal phenotypes of organ morphology, including organ morphology defects and left-right asymmetry defects, etc. Exogenous supplementation of migrasomes can significantly reduce the proportion of defects (Jiang et al., 2019).

Migrasomes can mediate the transfer of mRNA between cells: the mRNA is transferred to the recipient cells by the migrasomes (Jiang Y. et al., 2023), and the mRNA in the recipient cells can change the life activities of the recipient cells.

The regulation of cancer cells in the tumor microenvironment, such as migrasomes in pancreatic cancer cells, can induce an inhibitory immune microenvironment to promote tumor growth (Qin et al., 2022).

### 7.4 Relationship of migrasome to cellular stress, development, and disease states

Current studies have revealed the role of migrasomes in the development and progression of diseases (Figure 3B).

Studies targeting nanoplastic-induced abortion in women have shown that *in vivo* expression of ROCK1 can effectively rescue trophoblast cell migration/invasion and migrasome formation, thereby reducing abortion (Wan et al., 2024), suggesting that the reproductive toxicity of some environmental exposure may be mediated by inhibition of migrasome formation.

The study of migrasomes in metastatic tumors has been carried out (Figure 3C). Violently migrating glioblastoma cells have the potential to produce migrasomes (Lee et al., 2024). Migrasomes are formed by inhibition of autophagosome/lysosome fusion, which is important for the degradation of cellular cargo under stress conditions (Jiao et al., 2021). In addition, ER-associated proteins are abundant in the migrasomes and increased ER stress can generate more migrasomes in glioblastoma cells. However, reduced migration promotes the unfolded protein response, thereby enhancing cell death under stress conditions (Lee et al., 2024). Migrasomes have a stress-relieving function for brain tumor cells under ER stress conditions.

BMSCs-derived migrasomes containing dermcidin enhance LC3-associated phagocytosis of pulmonary macrophages and prevent post-stroke pneumonia, demonstrating dual functions of anti-infection and immunomodulation and superior therapeutic efficacy over antibiotics (Li et al., 2023).

The retinal pigment epithelium (RPE) can generate migrasomes in the microenvironment of proliferative vitreoretinopathy (PVR) (Wu et al., 2022). Since migrasomes can be internalized by RPE, they play a key role in the activation of RPE and the progression of PVR, increasing the migration and proliferation ability of RPE. TSPAN4 expression and migrasome production in such lesions are induced through the TGF-β1/Smad2/3 signaling pathway (Wu et al., 2022). Targeting TSPAN4 or blocking migrasome formation may be novel therapeutic approaches against PVR.

The migrasome and its element TSPANs are widely expressed in the cardiovascular system, and TSPAN8, TSPAN24, TSPAN12, and TSPAN29 are the major TSPAN family members that promote angiogenesis (Hemler, 2014; Bucher et al., 2017; Heo and Lee, 2020). Thus, TSPAN, migrasomes, and migrating cells may play important roles in regulating vascular homeostasis. Quantitative mass spectrometry has shown that monocyte migrasomes are enriched in proangiogenic factors and that purified migrasomes promote the capillary formation and monocyte recruitment *in vivo* (Zhang et al., 2022). Macrophage-derived migrasomes and subsequent complement activation are responsible for blood-brain barrier damage in cerebral amyloid angiopathy (CAA) (Hu et al., 2023).

## 8 Migrasomes are organelles rather than extracellular vesicles

Although migrasomes can also function as EVs, there are significant differences between migrasomes and other extracellular vesicles and can be used as a means of differentiation.

The most important distinction between microvesicles and other EVs is the mode of biogenesis. Microvesicles are generated by direct out-budding and fission through the plasma membrane (Cocucci et al., 2009), a process that results from a dynamic interplay between phospholipid redistribution and cytoskeletal protein shrinkage (Akers et al., 2013). Unlike migrasomes, exosomes, and microvesicles, which are secreted during normal cellular processes, apoptotic bodies are formed only during programmed cell death. They are also characterized by the presence of organelles within the vesicles (Akers et al., 2013). Membrane blistering is mediated in part by actin-myosin interactions (Coleman et al., 2001). Microvesicles and apoptotic bodies are obviously different from other EVs and can be identified.

There are a variety of proteins on the membrane of exosome vesicles, among which tetraspanin 4 (TSPAN4) and integrin family adhesion molecules are the most abundant. CD9, CD63, CD81, CD151, and tetraspanin 8 (TSPAN8), as adhesion molecules of specific tetraspanin families, participate in the biological processes of exosomes. Exosomes contain a variety of lipids, including cholesterol and sphingomyelin. In addition, exosomes also contain many RNA and DNA molecules, such as mRNA, microRNA (miRNA), long non-coding RNA (lncRNA), and mitochondrial DNA (mtDNA) (Xu et al., 2018; Kalluri and LeBleu, 2020). This is very similar to the migrasome components described above, including TSPAN4, cholesterol, integrin, mRNA, and microRNA (miRNA). Finally, using mass spectrometry, the researchers found that the two structures shared only about 27% of the protein, and the proportion of the material was not the same on their respective membranes (Zhao et al., 2019). It was finally demonstrated that although the migrasome is a membrane-wrapped vesicular structure similar to MVB, it lacks the surface marker of MVB, lysosomal-associated membrane protein 1 (LAMP1) (Ma et al., 2015). Exosomes are released from cells by fusion of MVBS with the plasma membrane (Kowal et al., 2014). Instead, the migracytosis involves translocation of cytoplasmic material into the migrasome, which is then released by disruption of RFs. Thus, migracytosis and exosome release are mechanistically distinct processes. We sorted out the differences between migrasomes and exosomes, including protein expression, production and release processes and biological functions, to clearly distinguish them (Table 1).

**TABLE 1:** Distinction between migrasomes and exosomes.

	Migrasome	Exosome
Diameter	0.5–3 µm	50–60 nm
Detach from the cell body	Ordered, marking the path of cell movement	spread rapidly and disorderly after leaving the cell
TSPANs expression profile	TSPAN4, TSPAN7, etc	TSPAN6, 8, 24, 25, 26, 27, 28, 29, 30, etc
Markers of membrane	TSPAN4, TSPAN7, ITGB1 (Integrin β1), ITGA5 (Integrin α5)	CD63, CD80, HSP60, etc
Only 27% of protein is the same	Four specific proteins: NDST1, EOGT, PIGK, and CPQ	Transmembrane proteins PGRL, LAMP1, LAMP2, CD9, CD63, CD81, CD151
Process of biogenesis	Formed by the assembly of large domains on the plasma membrane, cytoplasmic material is translocated into the migrasome and then released by the destruction of RFs	First produced as vesicles of multivesicular bodies (MVBs) and are released when MVBs fuse with the plasma membrane
Function	• Signal molecules, such as chemokines, cytokines, and growth factors, form local regional signal centers with the distribution of migrasomes • Maintenance of cellular homeostasis, such as removal of stressed mitochondria • Development of cells	Intercellular signaling, through membrane surface molecules bind directly or contents release

Migrosomes are membrane-bound cellular structures with characteristic morphological features that cells can use to release cellular contents, including vesicles and cytoplasmic proteins (Ma et al., 2015). In cell biology, organelles are specialized subunits of cells that perform specific functions. Another key feature of an organelle is that it is usually enclosed within its own membrane. Having a fixed membrane structure and being able to perform specific biological functions supports the idea that the migrasome is an organelle and not just a vesicular structure.

## 9 Discussion

As an organelle that can be released outside the cell, the migrasome exhibits an intercellular communication function through the nanoparticles released by its rupture (Figure 3). The specific source, composition, biological role, and final destination of these nanoparticles may hold the key to explaining certain physiological or pathological processes. As mentioned above, migrasomes have been found to play a role in bone and eye diseases, and have been proposed as potential targets for some tumors.

In terms of migrasome production, it has been found that PIP5K1A is recruited to migrasome formation sites before migrasome formation (Ding et al., 2023), and forms a possible trigger signal. It remains unknown how PIP5K1A is recruited to this particular site. In addition to integrins, there may be other effectors and adaptor proteins that contribute to migrasome biogenesis. Moreover, it is not clear how the activity of these proteins is regulated in the context of migrasome biogenesis.

There is a possibility of an association between autophagosome and migrasome formation in the maintenance of cellular homeostasis. It has been observed that inhibition of autophagosome/lysosome fusion to increase the number of autophagosomes can generate migrasomes capable of alleviating cellular stress (Lee et al., 2024) (Figure 3D). Complementation experiments of autophagy induction and autophagy-related genes are important to reveal whether autophagy and migrasome formation are related.

The observation in zebrafish is that migrasomes providing the CXCL12 chemokine are produced in both mesodermal and endodermal cells and that CXCL12 chemotaxis induces normal migration of dorsal first cells (DFC) (Jiang et al., 2019). Thus, it has been established that migrasomes play an important role in the development of organisms and that migrasomes are signaling organelles that can provide specific biochemical information to coordinate organ morphogenesis. The release of exosomes from cranial neural crest cells in the chick embryo promotes their directional migration and velocity (Gustafson et al., 2022), and monocytes deposit migrasomes rich in pro-angiogenic factors to promote angiogenesis (Zhang et al., 2022). More and more studies have focused on the role of migrasomes in embryonic development in different animals.

Other cells derived from the monocytic lineage, such as osteoclasts (OC), play key roles in bone development, bone remodeling, and fracture healing (Kylmaoja et al., 2016; Yahara et al., 2022). Studies of the timing and properties of RANKL-stimulated OC differentiation have revealed the appearance of migrasome-like vesicles along filopodia when mononuclear preosteoclasts approach and fuse with other preosteoclasts (Lampiasi et al., 2021). Migrasomes provide spatiotemporal information for cell communication during cell migration (Ma et al., 2015). These migrasomes generated during the migration of mononuclear pre-OC may also mediate the migration and fusion of pre-OC and subsequent OC differentiation (Lampiasi et al., 2021) (Figure 3D).

EVs carry proteins and nucleic acids derived from parental cells and can be used for early diagnosis of a variety of diseases (Drees et al., 2021; Zhang et al., 2021; Sun et al., 2022). Migrasomes that are detached outside the cell can play a role similar to that of EVs. Podocytes release “damage-associated” migrasomes during migration, and urinary migrasomes serve as potential diagnostic markers of early podocyte injury and are a more sensitive indicator of podocyte injury than proteinuria (Liu et al., 2020) (Figure 3B). Thus, similar to EVs, purification analysis of migrasomes may become a more intuitive indicator for clinical diagnosis of some diseases, or play an earlier role in suggesting diseases.

Tumor cells are mostly migratory and invasive, and the studies that have been carried out in glioblastoma have shown the broad prospects of migrasomes in the field of cancer, and the first pan-cancer analysis of migrasomes has found that they play an important role in tumor development and immune escape (Qin et al., 2022). High expression of migrasomes is associated with poor prognosis in cancer patients and may lead to poor patient survival (Qin et al., 2022). Therefore, aberrant migrasome expression may serve as one of the evaluation indicators for predicting pan-cancer in humans. In the case of pancreatic cancer, migrasome are rich in chemokines such as CXCL5 and cytokines such as TGF-β1, which can be released into the surrounding environment to recruit immune cells and induce their differentiation into immunosuppressive and carcinogenic phenotypes, further contributing to malignant biological functions and immune escape of pancreatic cancer (Zhang et al., 2020).

It was mentioned above that the migrasomes play a role by releasing intracellular material after rupture or phagocytosis. Proposed cancer nanodrug therapies utilize nanoparticles to form shells on RFs and migrasomes (Cheng et al., 2022), thereby blocking the recognition, endocytosis, and elimination of migrasomes by surrounding tumor cells, potentially erring tumor metastasis mechanically (Figure 3E).

Future studies will reveal the role of migrasomes in the disease microenvironment and immune process. As migrasomes can mediate intercellular material transfer like other EVs, migrasomes, as vaccine or drug carriers, become potential delivery agents of targeted drugs in the process of disease prognosis and immunotherapy, to achieve the effect of precision therapy (Figure 3F). Interestingly, unlike EVs, migrasomes display directional and chemotactic properties when they do their job, such as guiding immune cells along the correct path and direction. Migrasomes released by neutrophils guide CD8^+^ T cells to the site of influenza infection (Lim et al., 2015). This may suggest that the characteristics of attracting immune cells of migrasomes can be used to form new therapeutic ideas in tumors or other diseases.

Migrasomes have shown an important role in cell-to-cell communication of stem cells, and mesenchymal stem cells (MSCs) associated migrasomes have the property of chemotactic hematopoietic-derived cells (Deniz et al., 2023), affecting embryonic development, and may provide new insights into disease therapy. However, whether MSCs-associated migrasomes affect the migration and/or retention of metastatic cancer cells in the bone marrow and thus constitute potential targets for cancer therapy remains to be evaluated. At the same time, it remains to be seen whether the disease microenvironment can affect the formation and role of migrasomes. Finally, it remains to be evaluated whether MSCs-associated migrasomes, like MSCs, have therapeutic uses in post-transplant host tissues, such as immunomodulatory properties.

## 10 Conclusion

In summary, our article reviews the facts that have been found in migrasome studies (Table 2). As a kind of organelle, migrasomes play an important role in cell homeostasis. In addition, intercellular communication is crucial in multicellular organisms. As a kind of organelle widely present in migrating cells, it has profound significance in revealing how cells interact with each other. Recent studies have shown that migrasomes play an important role in the physiological process of the body, the occurrence and development of diseases, as well as the diagnosis and prognosis of diseases. Several methods for isolation, identification, and observation of migrasomes have been proposed. Given the widespread existence of cell migration in the physiological and pathophysiological processes of the body, we believe that continued research on the migrasome will reveal its role in more fields in the future.

**TABLE 2:** Factors found in migrasomes

Migrasome	Known facts
Diameter	0.5–3 µm
Detach from the cell body	Ordered, marking the path of cell movement
Process of formation	Nucleation, maturation, and expansion
Cytoskeleton	F-actin, α-tubulin and vimentin intermediate filaments
Release	Formed by the assembly of large domains on the plasma membrane, cytoplasmic material is translocated into the migrasome and then released by the destruction of RFs
Biomarkers	Four specific proteins: NDST1, EOGT, PIGK, and CPQ Markers of membrane: TSPAN4, TSPAN7, ITGB1 (Integrin β1), ITGA5 (Integrin α5)
Means of observation	• Transmission electron microscopy • Scanning electron microscopy • Long-term high-speed high-resolution 3D imaging: 2pSAM • Better spatiotemporal resolution and lower phototoxicity: DAOSLIMIT
Function	• Releasing signaling molecules • Maintaining cell homeostasis • Development of cells • Indicating disease progression
